# Some Facts and Fiction That Will Stimulate Atlanta Doctors to Activity in Medico-Social Refirm

**Published:** 1915-11

**Authors:** C. C. Aven


### SOME FACTS AND FICTION THAT WILL STIMULATE ATLANTA DOCTORS TO ACTIVITY IN MEDICO-SOCIAL REFORM.

By C. C. Aven, M. D.

Presenting a paper of this character, even to an interested audience, is not an altogether pleasant task, for some one will assume that the man is a crank or a fanatic that advances ideas in a field where no attention has been directed to this particular subject. Nevertheless, I am attempting to call your attention to some facts we should know and must begin to recognize as realities.

Have we heretofore as physicians made ourselves aware of the fact that we are inefficient, non-aggressive and too ready to stop short of accomplishing real good to humanity, or in other words, undermining the cause?

The knowing of symptomatology is knowledge gained by careful, accurate study of an existing condition and should we sit idly by and be content to study the effect of a cause? No, we would be little short of dereliction. Now, a careful, accurate study of the cause of disease calls for the co-operation of other professions. Would it be a little farfetched to make the statement that we as physicians are filled with petty jealousies that make us too narrow to want some other profession to share the distinction and honor of being a co-worker in such a great cause as preventive medicine. I am afraid that is true.

Our professional allies, the Social Workers, are new in this section of the country, but we will soon come to depend on their aid for better work among our own. Some one has said "In order to understand the 'real trouble' of a patient, you the doctor, must know

1. Bodily state, physical condition, diagnosis, prognosis, etc;
2. Mental state;
3. Bodily environment, work, housing, food, etc.;
4. Mental environment,—influence (good or bad) of family, friends, etc."

Can the physician ascertain these factors in the patient's illness? Yes, but not alone, and properly and scientifically to treat such cases he must know all these things. Can the average nurse in her casual way get the facts? No, she is trained, but only medically and not sociologically. Therefore, we need the services of an individual with these two things linked together in one person, the Social Nurse.

The essential points in the treatment of disease are two:

1st. Careful study of bodily state and cause of such by physician.

2nd. A sympathetic (note the word) study of the individual patient in his home.

In our modern times we are drifting more to specialization and rightly so, for no opthalmologist attempts to remove an ovary or appendix. Social work is a field for specialization, for there are problems confronting the medical profession to-day that truly demand a specialist in social work.

We have only recently discovered that pellagra is probably a disease of nutrition and environment, and we have known for some time that tuberculosis was a disease of nutrition, housing conditions and home life: that the treatment of such diseases as diabetes is almost wholly a dietetic one, and we as leaders of public health and hygiene must get busy and bring about a solution of this great problem.

The crying need therefore is for the physician to demand a trained worker to see that his diabetic case gets the proper diet after leaving the hospital, or to see that his tubercular case is making the very best of his environment. So truly speaking, preventive medicine simmered down to a practical point comes to the problem of the home.

We all admit that the Hospital is the centre from which emanates the educational work of preventive medicine, but the difficulty arising is that the education does not continue after the patient leaves the hospital, hence the necessity for a follow-up system for these cases. What are the results of our present system? Many, but a few are as follows:

A case of an infant, has an illeo-colitis in summer as result of improperly prepared food, etc.; sent to hospital, cured from the view point of the hospital, but the cause, the ignorance or carelessness of the mother has not been corrected and baby returns shortly with recurrence.

A case of diabetes in, we will say, a non-American patient; remains in hospital till sugar disappears from urine. He is given a diet list, few instructions as to mode of living, etc., but his race customs have not been studied and he soon returns. Therefore, we ought to know what they can be induced to eat under ordinary conditions before we can do anything intelligently with the problems of diet in disease. This is a home problem and deals with social investigation.

Many such instances could be related, but I shall offer some suggestions as to the remedy.

1st. Prove conclusively by statistics and other means that this question of medico-social investigation involves economics, showing the actual value of dollars and cents; and this can be done by demonstrating that you are dealing with facts and not with mere theories.

2nd. Demand the workers as they are an essential part of your treatment of disease, and when this is done you create interest first among ourselves as physicians, second, among those that support, endow and in other ways maintain our hospitals.

After you have done this and have workers what has happened?

1st. Humanity as a whole is bettered;

2nd. The doctor is a better one;

3rd. The patient gets the best results;

4th. Life is prolonged and the actual earning capacity of every member of society is increased;

5th. The hospitals' burdens are lessened by relapses, recurrences, etc.;

6th. Better vital statistics may be obtained.

In closing let me ask the hearty co-operation of every one present in promoting interest in this movement of Medico-Social Reform and in a few years we may predict phenomenal success.

THE FENCE OR THE AMBULANCE.

'Twas a dangerous cliff, as they freely confessed,
  Though to walk near its crest was so pleasant,
But over its terrible edge there had slipped

A Duke and full many a peasant.
So the people said something would have to be done,
But their projects did not at all tally,
Some said "Put a fence round the edge of a cliff,"
Some, "An ambulance down in the valley."

But the cry for the ambulance carried the day,
For it spread through the neighboring city;
A fence may be useful or not, it is true,
But each heart was brimful of pity,
For those who slipped over that dangerous cliff;
And the dwellers in highways and valley
Gave pound and gave pence, not to put up a fence,
But an ambulance down in the valley.

"For the cliff is all right if you're careful," they said,
"And if folks even slip or are dropping
It isn't the slipping that hurts them so much
As the shock down below when they're stopping."
Then an old sage remarked, "It's a marvel to me
That people give far more attention
To repairing results than to stopping the cause
When they'd much better aim at prevention."

"Let us stop at its source all this mischief," cried he.
"Come, neighbors and friends, let us rally,
If the cliff we will fence we might almost dispense
With the ambulance down in the valley."
"Oh, he's a fanatic," the others rejoined.
"Dispense with the ambulance? Never!
He'd dispense with all charities, too, if he could;
But no! We'll protect them forever.
Aren't we picking up folks just as fast as they fall?
And shall this man dictate to us? Shall he?
Why should people of sense stop to put up a fence
While their ambulance works in the valley?"

But a sensible few, who are practical, too,
  Will not bear with such nonsense much longer;
They believe that prevention is better than cure
  And their party will soon be the stronger,
Encourage them, then, with your purse, voice and pen,
  And (while other philanthropists dally)
They will scorn all pretense and put up a stout fence
  On the cliff that hangs over the valley.

